# Mental health pharmacists views on shared decision-making for antipsychotics in serious mental illness

**DOI:** 10.1007/s11096-016-0352-z

**Published:** 2016-07-23

**Authors:** Mediha Younas, Eleanor Bradley, Nikki Holmes, Dolly Sud, Ian D. Maidment

**Affiliations:** 1 Aston University, Aston Triangle, Birmingham, B4 7ET UK; 2 Institute of Health and Society, University of Worcester, Henwick Grove, Worcester, WR2 6AJ UK; 3 Forensic Services, Nottinghamshire Healthcare NHS Foundation Trust, Rampton Pharmacy, Rampton Hospital, Retford, Nottinghamshire DN22 0PD UK; 4 Leicestershire Partnership NHS Trust, Glenfield Hospital, Leicester, LE3 9EJ UK; 5 School of Life and Health Sciences, Medicines and Devices in Ageing Cluster Lead, Aston Research Centre for Healthy Ageing (ARCHA), Aston University, Aston Triangle, Birmingham, B4 7ET UK

**Keywords:** Antipsychotic prescribing, Mental health pharmacy, Shared decision making, United Kingdom

## Abstract

*Background* People diagnosed with serious mental illnesses (SMIs) such as schizophrenia and bipolar affective disorder are frequently treated with antipsychotics. National guidance advises the use of shared decision-making (SDM) in antipsychotic prescribing. There is currently little data on the opinions of health professionals on the role of SDM. *Objective* To explore the views and experiences of UK mental health pharmacists regarding the use of SDM in antipsychotic prescribing in people diagnosed with SMI. *Setting* The study was conducted by interviewing secondary care mental health pharmacists in the UK to obtain qualitative data. *Methods* Semi-structured interviews were recorded. An inductive thematic analysis was conducted using the method of constant comparison. *Main outcome measure* Themes evolving from mental health pharmacists on SDM in relation to antipsychotic prescribing in people with SMI. *Results* Thirteen mental health pharmacists were interviewed. SDM was perceived to be linked to positive clinical outcomes including adherence, service user satisfaction and improved therapeutic relations. Despite more prescribers and service users supporting SDM, it was not seen as being practised as widely as it could be; this was attributed to a number of barriers, most predominantly issues surrounding service user’s lacking capacity to engage in SDM and time pressures on clinical staff. The need for greater effort to work around the issues, engage service users and adopt a more inter-professional approach was conveyed. *Conclusion* The mental health pharmacists support SDM for antipsychotic prescribing, believing that it improves outcomes. However, barriers are seen to limit implementation. More research is needed into overcoming the barriers and measuring the benefits of SDM, along with exploring a more inter-professional approach to SDM.

**Electronic supplementary material:**

The online version of this article (doi:10.1007/s11096-016-0352-z) contains supplementary material, which is available to authorized users.

## Impacts of findings

- Pharmacists can play a key role in developing a shared decision making model for antipsychotic prescribing.
- Greater efforts to engage service users and multidisciplinary team working are required to enable SDM to be implemented.
- Further research is required on the views of service users on SDM and the role of pharmacy in supporting SDM.

## Introduction

At least one in four people in the UK will experience a mental health problem and up to two percent of the population will be diagnosed with a serious mental illness (SMI) during their lifetime [1]. For the purposes of this research SMI is considered to include diagnoses that are treated with antipsychotics, typically schizophrenia and bipolar affective disorder [2]. The adverse effects of antipsychotics can decrease adherence rates [3].

Adherence, the currently accepted term used for medication-taking behaviour, is defined ‘as the extent to which the patient’s action matches the agreed recommendations’ [4]. Adherence presumes agreement about the proposed medication, between the prescriber and the person taking the medicine, emphasising the importance of shared decision-making (SDM; [4]). A third to a half of all medications for long-term conditions are not taken as recommended, and treatment adherence is one of the biggest challenges in mental health [4, 5]. Adherence in SMI is very poor; estimated non-adherence rates for people diagnosed with schizophrenia range from 40 to 75 % [6, 7]. Studies have found that 75 % of people with chronic schizophrenia discontinue their medication within 18 months [8] and non-adherence rates in bipolar disorder range between 20 and 60 % with a mean of 41 % [9, 10]. Antipsychotic prescribing lends itself to SDM, because the adverse event profile is the main factor in the choice of antipsychotics [11].

SDM is defined by the NHS as ‘the conversation that happens between a patient and their healthcare professional to reach a healthcare choice together’, where both parties consider what is important to the other when selecting treatment. There are ethical, clinical and economic arguments for SDM [12]; it represents a method of healthcare communication that promotes patient-centred care and sharing expertise between clinicians and service users [13, 14]. The most accepted model is that of Charles and colleagues, which emphasises patient autonomy, informed consent and empowerment [15]. SDM is founded on partnership and opposed to a paternalistic model of healthcare [16]. A recent Department of Health White Paper stated that ‘care should be personalised to reflect peoples’ needs, not those of the professional or the system’ and patients should be involved in treatment decisions [17]. People diagnosed with SMI can be fully engaged with making decisions and seek a more collaborative approach, thus treatment decisions should be made by the service user and the healthcare professional working together and considering both the likely benefits and possible adverse effects of the medication [11, 18].

SDM has been linked to improved quality of care and service user satisfaction [19, 20]. However, the evidence base supporting the use of SDM for chronic conditions, notably mental health [21] and the use of SDM for decisions made on multiple occasions over the longer-term is limited. Hamann [22] found that SDM increased knowledge and perceived involvement in treatment in inpatients with schizophrenia. However, SDM failed to show long-term benefits in the same study [23]. A Cochrane review found that no conclusions could be drawn regarding the effectiveness of SDM interventions for people with mental health problems and highlighted the urgent need for more research [13]. A more recent study found that although a pharmacist intervention based on SDM significantly improved adherence, treatment satisfaction and beliefs about medication in people with depression, it had no significant effect on depressive symptoms [24].

Both service users and clinicians appear to support SDM [25]. However, only 32 % of service users report that their views about treatment were considered ‘to some extent’ and less than half (43 %) were informed about adverse effects, suggesting clinicians are not engaging in SDM [26]. The lack of a multi-disciplinary approach and the perceived difficulty of implementing SDM with service users who may lack insight are barriers to SDM across mental healthcare [21, 27]. In addition, there are structural obstacles to collaborative care in psychiatry which include timely access to relevant, reliable clinical information, and therefore research is vital to understand the practicalities of SDM in practice [21, 28, 29].

Whilst experiences of and attitudes of consultant psychiatrists towards shared decision making in antipsychotic prescribing have been studied, qualitative data on the views of other key groups of healthcare professionals involved in medication management across mental health services, including pharmacists, is lacking [21]. This study aimed to understand the views and opinions of mental health pharmacists in the UK who are increasingly developing clinical roles. These clinical roles include; advising prescribers and clinicians on the most appropriate medication after interviewing patients; patient education and advocacy; attending and directly inputting into multi-disciplinary meetings. These roles are generally independent from the prescribing process although a limited number of pharmacists may have a caseload with a prescribing role.

## Aim

To elucidate the experiences and opinions of mental health pharmacists about implementing SDM in the process of antipsychotic choice and prescribing in SMI.

### Ethics approval

The project received approval from the Aston University Ethics Committee.

## Methods

### Design

An exploratory qualitative study design that followed COREQ (Consolidated Criteria for Reporting Qualitative studies) guidelines [30] was employed.

### Participant recruitment and consent

Mental health pharmacists with a minimum of 12 months experience in mental health pharmacy practice were recruited, on the basis that they were more likely to have an understanding of SDM and be undertaking advanced clinical roles. Participants were recruited from the Midlands region of the UK. Initially convenience sampling was used; known contacts meeting the inclusion criteria were identified [31]. Further participants were recruited through active snowballing [32]. Potential participants were emailed with the project aims and participation requirements. Written informed consent was obtained prior to participation.

### Inclusion criteria

Mental health pharmacists with a minimum of 12 months experience in mental health pharmacy practice.

### Interview structure and collection

A semi-structured interview was used to allow the interviews to be participant-led and participants to express their views openly [33–35]. An initial interview topic guide, based on the literature on research into SDM in mental health, was constructed to focus the interviews [36]. This guide was reviewed and amended by the academic supervisor (IM) and two practising mental health pharmacists (NH, DS; see “Appendix 1” of Supplementary Material). The schedule was adapted following each interview, using an iterative approach [37, 38]. Participants were given a chance to provide feedback and suggest questions to be included in the topic guide. Eleven face-to-face and two phone interviews were conducted; each lasted between 20 and 35 min. These 13 interviews were deemed sufficient to provide the necessary identification of themes. The transcripts were reviewed after each interview and data saturation was perceived to have been met as no new themes were identified in the last set of interviews [39]. Interviews were audio recorded and a verbatim account produced from these recordings [40]. The recordings were checked against the transcripts several times [41].

### Data analysis

Thematic analysis, based on the identification of themes, was conducted by MY [34]. The transcripts were independently reviewed by IM; any disagreements on the coding scheme were resolved by discussion between IM and MY.

The constant comparison method informed by grounded theory was used whereby the data analysis takes place alongside data collection [42, 43]. Each interview was reviewed before the next commenced to identify emerging patterns in the data and assist structuring of further interviews [33]. Coding took place in three stages [35, 44], as follows:

- Open coding was used to identify themes; coding and categories were refined.
- Axial coding was then used. Extracts were photocopied from the original data and arranged with the codes together in files.
- Selective coding was used; data was analysed and re-organised. Themes were arranged according to their relation to the research question.

### Reflexivity

Qualitative research as a process necessitates and acknowledges the key role of reflexivity, and the important role played by any researcher’s background, perceptions and interests in the topic [30, 33, 36, 37]. Within this study, the interviews were conducted by a female pharmacy undergraduate student of Indian sub-continent descent. In preparation for the study, the student received training in research methods including qualitative research, supervisory guidance during the development of the interview schedule and support from the research team in relation to the interpretation and analytic process.

## Results

Fifteen participants were recruited but two interviews didn’t take place due to time constraints. Of the 13 participants interviewed the majority were aged between 30 and 40 years old (six of the 11 participants who reported this information). Ten participants were female and three were male. Four main themes were identified: attitudes to SDM; barriers to implementation; benefits of SDM; and the role of mental health pharmacists.

### Attitudes to shared decision making

#### Pharmacist attitudes

Almost all the pharmacists felt SDM was a positive concept and supported its use in antipsychotic prescribing.

I totally support the idea…they’re powerful drugs therefore….patients should have the opportunity to articulate what factors are most important to them and this should be taken into consideration when choosing treatment. (In01)

The complexity of antipsychotic use was recognised, with reference in particular to side effects and the impact on adherence rates. For these reasons, patient choice was highlighted as being particularly important:

The choice should be dependent on what the patient will tolerate in regards to side effects. (In13)

The pharmacists believed that it was important to involve service users in the discussion, even if agreement could not be reached.

We get that quite a lot. We have involved them in the treatment plan but they might not agree still with the decision that we have tried to involve them with. (In06)

However, some pharmacists viewed SDM as a tool to achieve adherence, to persuade the patient to take the medication, rather than an agreement negotiated between two equal parties.

A few cases it has helped but we still need to persist in getting them to take their medication so it’s still an issue. Once they realise that medication is important they feel better then hopefully…sometimes when we have given them a lot of choice the patient seems to change their mind a lot. (In06)

#### The views of pharmacists on the attitudes of prescribers

Pharmacists believed that attitudes towards SDM amongst prescribers were variable.

There’s a very broad church amongst (prescribers)…some are excellent and some have the view the patients should do as they’re told. (In04)

The majority, however, felt that there had been a positive cultural shift, with attitudes moving towards greater service user involvement.

Attitudes have definitely changed in my 16 years in mental health, early on it was very much…I’m the doctor and this is what’s right. I think health as a whole has shifted…engaging with the patient a lot more. At one time…it was…if you tell patients about side effects they won’t take the medication. (In03)

However, some pharmacists felt that SDM was not practised as widely as it should be due to the perceived difficulties in relation to patient engagement:

I’m not saying…they don’t want to involve patients but I think it’s because of the difficulty of engaging patients. (In11)

#### The views of pharmacists on the attitudes of service users

The pharmacists also believed that the attitudes of service users towards SDM were variable; some service users were seen to want involvement in the decision-making process whereas others preferred the clinicians to make the decisions.

Some patients want to be told what to do; other patients want…to make the decision themselves. (In04)

There was, however, a general consensus amongst the pharmacists that service users, particularly younger service users, were increasingly wanting to be involved in the decision-making process and have more choice, partly due to changes in society.

They crave that involvement and…empowerment…in a largely consumerist society people want and expect choice and…more autonomy. (In01)

### Barriers to implementation of SDM

#### Capacity and insight

A lack of service user insight was seen by the participants as an obstacle to SDM.

If they don’t have insight…it doesn’t matter what decision you make or information you give (them)…(if they believe that) there’s nothing wrong with them they don’t need to take treatment. (In04)

Several pharmacists highlighted the fact that when treatment decisions (initiation, dose change or switching) are frequently made, that service users are often acutely unwell and so these are times of difficulty in relation to SDM. Moreover, if they are detained under the mental health act then treatment decisions may be imposed on the service user as being in their best interests rather than attempting to overcome the barriers associated with SDM at these points:

They might be acutely unwell…they might not be in a position to make a decision they might be forced to have treatment against their wishes so in that scenario you’re not going to be able to provide them with SDM. (In03)

When medication regimes were working well, there was often hesitancy from clinicians to make changes.

Switching a treatment when they have been stabilised a long period of time is actually a very scary thing to do. (In13)

However, the majority of pharmacists felt that SDM could be implemented with most of the service users, most of the time:

If you are flexible in your approach…but nevertheless you can still have some degree of conversation to enable them to be a part of the SDM process the vast majority of the time. (In07)

#### Time

Time was a key barrier to SDM. Pharmacists believed that clinicians often did not have the opportunity to speak to service users or time to fully implement techniques of SDM:

It takes a lot longer than just writing a prescription. (In06)

Such time pressures were increasingly problematic with services experiencing high demand:

There’s always a demand for beds, it does have an impact on SDM. (In05)

Not having the time in outpatient clinics. (In13)

### Potential benefits of SDM

#### Adherence

Pharmacists felt that if service users were genuinely involved in the prescribing decision, this could improve adherence.

If they’re taking part in the decision they have an interest in the outcome…if you don’t involve them and you are imposing something, as soon as they go out of the door they won’t actually be interested in continuing with it. (In05)

Mental health was viewed as similar to any other chronic illness management in that giving more autonomy to service users improved adherence to medication.

I think it’s like any other condition…the more autonomy you give the patient…the more likely they are to comply. (In10)

Importantly, the absence of SDM was believed to result in non-adherence and high rates of re-admission to hospitals.

It’s not as high as it ought to be otherwise…they wouldn’t have so many patients relapsing, we have these revolving door patients that keep coming in again and again, people just don’t take their medication. (In02)

However, there was recognition that those service users who were engaged and interested in SDM could be those who were more likely to be adherent regardless of approach.

Those patients who can actually engage are more likely I think to actually be concordant. (In11)

#### Service user satisfaction

Service users were said to respond well to SDM, and appreciate being involved in decisions about their care, improving the therapeutic alliance. One pharmacist who believed that SDM had a positive effect on the therapeutic alliance quoted one service user saying:

You were one of the few people who saw me as a human being and gave me a choice, when everyone else was just telling me what to do. (In07)

SDM could help service users feel more valued and respected, and work towards removing some of the stigma that is associated with mental health.

It’s huge stigma all around… so if you treat them like every other human being… they’re going to feel valued and respected definitely …there’s definite improvement, they feel at the centre of their care…they will respect you for giving them that rather than being domineering and telling them…. I know better than you. (In02)

### The role of the mental health pharmacist

#### Service user counselling

The pharmacists felt that service users were often more open about medication issues with them than other health professionals, particularly about sensitive side effects such as sexual dysfunction.

I’ve had a patient discuss sexual dysfunction with myself……where they didn’t discuss it on the ward review because they felt embarrassed to talk about it with the consultant. (In13).

Pharmacists felt they were often seen as an independent person compared to the prescriber and therefore able to have an open conversation with service users about medication.

I do think we’re in a very good position to discuss things because we are….seen as independent. (In10)

More research into the impact pharmacists can have upon clinical outcomes such as relapse rates was suggested.

I think we could reduce (the) relapse rate. Somebody needs to do a study into pharmacist input….and the impact it has on non-concordance. (In13)

#### Multi-disciplinary team (MDT) working

The level of input that mental health pharmacists have in SDM was dependent on the leadership of the MDT, with some clinical teams more collaborative than others, and resourcing within pharmacy services.

Some of the clinical teams I’m in are very collaborative and very collegiate… I’ve also worked in teams where there’s very little conversation apart from between nurses and doctors, me as the pharmacist has to almost fight to say something. (In07)

Mental health pharmacists clearly felt that they had more to offer and were often underutilised.

I think they have a really difficult job, but if they let us help them, a bit more in recognising we have a resource here, that we can actually use that we have the knowledge. (In11)

Pharmacists believed that a more inter-disciplinary approach with a referral system could support their involvement in SDM.

Some way of referring patients to a pharmacist clinic….but there’s no actual referral process (In13)

## Discussion

Pharmacist participants were supportive in principle for SDM, particularly when considering the use of antipsychotic medication, and believed that practising SDM was a key part of stigma-free clinical care. Like previous research, the pharmacists felt SDM increased service user satisfaction, which in turn improved the therapeutic relationship and was key to achieving long term treatment success and positive outcomes by improving adherence to medication [19, 45–50].

The pharmacists perceived that attitudes of both services users and prescribers to SDM varied. Some pharmacists felt that a minority of service users were happy with the clinician making treatment decisions on their behalf. Other research has also identified this group who believe ‘the doctor knows best’; perhaps because they undervalue their expertise in relation to clinicians and want to be ‘a good patient’ [47, 51]. Most service users, however, particularly those in younger age groups, were said by the pharmacists to increasingly crave involvement, which is in line with previous research [27, 46, 52]. This change may reflect an increasingly consumerist society, where choice is expected [53–55].

A strong, trusting relationship, with health care professionals and service users both accepting an active role, is essential to the success, or otherwise of SDM [47]. Yet service users often describe mixed feelings, that they are both helped and misunderstood by healthcare professionals, and commonly report experiencing discrimination [56, 57]. SDM involves the clinician respecting the right of service users to make treatment decisions, even if they disagree with this decision [58]. However, like other research, we found a mixed picture; the pharmacists perceived that some prescribers adopted an authoritative approach, dominating consultations and failing to take into account the views of service users [26, 59–62].

The participants perceived a lack of service user insight as the main barrier to SDM. Service users suffering from acute illness were said to lack capacity precisely when medication was most likely to be initiated or changed and, therefore, when SDM was important. However, when the illness being treated was well controlled, and the service user may be more likely to be able to be engaged in SDM, the pharmacists perceived that clinicians would be reluctant to change medication due to concerns about the illness becoming less well-controlled.

Generally the pharmacists reported that SDM was not possible with service users treated under the mental health act without their consent [63]. This act is designed to protect the rights, health and safety of people with a mental health disorder and the safety of others; it covers the circumstances in which someone can be detained for treatment [63]. Unlike some other studies, some pharmacists in this study did not view capacity in absolute terms [21]. They felt more should be done to engage service users and that SDM should be attempted with all service users to varying degrees depending on the level of insight and capacity. This echoes other research, which has found that service users with SMI value the opportunity to collaborate with those providing their care and are prepared to engage with SDM within the current patient-professional relationship [47]. SDM can also improve treatment knowledge amongst service users with schizophrenia potentially reducing the risk of medication errors [18, 64–66].

However, rather than focus on individual barriers, it may be more relevant to consider structural barriers to SDM in mental health practice such as a lack of time, poor communication between clinicians and service users, and limited access to evidence-based information [28, 58]. SDM can be seen to be a time consuming activity to undertake [22, 27]. In this research pharmacists reported the lack of time of both pharmacists and prescribers to be a barrier, with pharmacists identifying that other duties were seen to override SDM; other research has found that lack of time is a commonly reported barrier by both health professionals and service users [27, 51, 59, 60].

The pharmacists felt they were able to play a vital role in SDM partly because their independence from the prescribing process enabled them to engage in SDM. Previous research has identified the need for an inter-disciplinary approach involving autonomous clinicians to engage service users in SDM [67–70]. However, many of the pharmacists felt that they did not always get the opportunity to be involved in the SDM process due to the lack of a structured referral system and multi-disciplinary approach or resources issues within pharmacy departments.

### Implications of study

Services should be structured to support SDM with a more inter-disciplinary approach. This could include a formal referral system to pharmacists or implementation of pharmacist clinics. Training for pharmacists (and potentially other clinicians) should highlight that SDM should be adapted depending on the state of illness at the time, but not abandoned.

### Further study

Further qualitative research on SDM, and more specifically the potential role of pharmacy, involving pharmacists, other clinicians and service users is required. Research is also required on the impact of SDM on outcomes including adherence to medication [19, 48–50, 71]. Future research should investigate whether clinicians use SDM differentially depending on various characteristics including how long they have known the service user for and what the medication is being utilized for. It could also cover service users’ views on the role of family members as advocates. Previous research has identified a role for healthcare professional ‘coaches’ not involved in treatment to actively support service users in engaging in SDM [58]. Therefore, future research could investigate the impact of ‘pharmacy medication management coaches’ on key outcomes.

### Limitations

All the participants recruited for the study came from the Midlands region and may not be broadly representative of attitudes and experiences of mental health pharmacists nationally and internationally. Moreover, we cannot be sure how long the participants had worked in mental health for (other than more than 1 year), whether they have a formal mental health qualification or their area of practice. We relied on convenience and snowballing sampling and relatively small sample sizes; however we found data saturation with consistent themes identified and no new themes identified in the last set of interviews. Additionally, identifying participants via known contacts may have influenced the interview responses in relation to socially desirable responses.

This research project only sought the views of mental health pharmacists; a future project should triangulate the data collection methods and also interview other clinicians and more importantly service users. Pharmacists are increasingly becoming prescribers and therefore future research should also compare and contrast the views and experiences of prescribing and non-prescribing (who are independent from the prescribing process) pharmacists.

## Conclusion

In keeping with previous research in this area, SDM was seen as a positive concept by the mental health pharmacists interviewed. SDM should take into consideration the service user’s ability to tolerate adverse effects and their preferences regarding medication. The pharmacists believed that such an approach could improve service users’ satisfaction with medication management services and ultimately adherence to medication. The pharmacists perceived that the attitudes of prescribers and service users, although noted as variable, to be increasingly in favour of SDM.

The pharmacists identified that the use of SDM was limited by barriers, particularly the difficulties perceived by clinicians of engaging people with SMI who lack insight and mental capacity in the process. Greater effort is seen to be needed to work around these issues and try to engage service users as much as possible. Structural issues, such as time pressures may also limit the use of SDM.

Pharmacists clearly feel they can play a vital role in SDM but their skills and knowledge in this area are being underutilised, limiting their opportunity to contribute. SDM is clearly seen as one way to improve outcomes, and more research on how it can be effectively implemented in mental health is required.

## Electronic supplementary material

Below is the link to the electronic supplementary material.

Supplementary material 1 (DOCX 17 kb)
