# Supplementary material for: Mental health pharmacists views on shared decision-making for antipsychotics in serious mental illness
Source: Int J Clin Pharm. 2016 Jul 23;38(5):1191–9. doi: 10.1007/s11096-016-0352-z (PMC5031729; doi:10.1007/s11096-016-0352-z)
Supplement: Supplementary file 1 — Supplementary material 1 (DOCX 17 kb) [file 11096_2016_352_MOESM1_ESM.docx]

# Appendix I

**Interview topic guide**

I am interested in the use of shared decision making in antipsychotic prescribing and will ask you some questions around this theme.

In general (within pharmacy) what does the term SDM mean to you? What would you say shared decision making is?

How do you feel SDM works in antipsychotic prescribing? – Is this something you feel you practice? (What kind of decisions do you involve the patient in can you give some examples of this)?

If so how regularly would you say you practice SDM?

What are your general thoughts about SDM?

1. Potential questions for Exploring the outcomes of using SDM when prescribing antipsychotics:

- In your experience what is the patient response to SDM? How do patients react to being involved in the SDM process?
- What difference if any do you think SDM has on the patient being prescribed antipsychotic in comparison to general prescribing?
- When you have practiced SDM, has there been any difference in the patient’s level of engagement in their healthcare?
- Would you say there is there any impact of SDM on patient adherence? How do you think it impacts medication adherence?

2. Potential Questions for exploring any risks associated with SDM:

- What are the attitudes of patients who have been involved in SDM in antipsychotic treatment regarding other aspects of healthcare/future treatment?
- Do you feel the prescriber’s competency will be questioned if they give the patient more choice?
- How appropriate do you feel SDM is? (Is it appropriate for all patients?) Are they any special considerations for certain at risk groups?
- Risks in general? — Further explore any risks mentioned.

3. Potential Questions exploring the problems with SDM use in practice:

- What is your experience with using SDM with patients who lack mental capacity or have limited insight?
- How is SDM for patients affected by having a multi-disciplinary team? (Having lots of people involved in one person’s treatment or care)
- How well does the multi-disciplinary team work in participating with SDM?
- Do you feel there are resource complications (time and money) for short term and long term implementation of SDM?
- Prescriber: What effect does carrying out SDM have on the prescriber? / What difference does it make to the prescriber? Give them a decision in comparison to making the decision solely? In terms of work load, practicality, ease of implementation?
- Generally what are prescriber attitudes to SDM?

4. Potential questions to explore relationships:

- How far does the clinician- patient relationship have an impact on SDM?
- How do you feel the patient-clinician relationship differs when SDM is involved compared to when it’s not involved Relationship long term treatment? (Before and After).

5. Potential Questions to explore the Overall Opinions of SDM:

- Amongst pharmacists prescribing antipsychotics do you see SDM being practised regularly?
- From your own experience have you in practice seen SDM make any difference in clinical outcomes in comparison to general prescribing?
- What do you think the role of mental health pharmacists is in SDM?/ Do you think that the MH pharmacist can practice SDM/ are in a good position SDM?
- Overall are you in favour of SDM? / National guidance encourages the use of SDM in antipsychotic prescribing do u support this?
- How can we encourage SDM use in practice?
